# Homeostasis and Cancer Initiation: Organoids as Models to Study the Initiation of Gastric Cancer

**DOI:** 10.3390/ijms23052790

**Published:** 2022-03-03

**Authors:** Sulaimon Idowu, Paul P. Bertrand, Anna K. Walduck

**Affiliations:** STEM College, RMIT University, Melbourne, VIC 3000, Australia; sulaimon.idowu@rmit.edu.au (S.I.); paul.bertrand@rmit.edu.au (P.P.B.)

**Keywords:** gastric cancer, organoid, gastric homeostasis, *Helicobacter pylori* infection

## Abstract

Gastric cancer represents a significant disease burden worldwide. The factors that initiate cancer are not well understood. Chronic inflammation such as that triggered by *H. pylori* infection is the most significant cause of gastric cancer. In recent years, organoid cultures developed from human and animal adult stem cells have facilitated great advances in our understanding of gastric homeostasis. Organoid models are now being exploited to investigate the role of host genetics and bacterial factors on proliferation and DNA damage in gastric stem cells. The impact of a chronic inflammatory state on gastric stem cells and the stroma has been less well addressed. This review discusses what we have learned from the use of organoid models to investigate cancer initiation, and highlights questions on the contribution of the microbiota, chronic inflammatory milieu, and stromal cells that can now be addressed by more complex coculture models.

## 1. The Need to Understand the Complex Factors That Lead to Gastric Cancer

In 2020, gastric cancer was the sixth most common carcinogenesis in males and females with a prevalence of 11.1/100,000 population (GLOBOCAN analysis [1]). Mortality remains high, and gastric cancer (GC) is the fifth most common cause of cancer death. 

Gastric cancer is heterogeneous, and can be broadly classified as intestinal or diffuse type, reflecting histological changes observed in established cases. The diffuse type is more often related to rare genetic abnormalities [2]. The intestinal-type GC is related to environmental factors; including diet, smoking, *Helicobacter pylori* infection or Epstein Barr virus (EBV) infection. Gastric carcinomas are also classified according to their anatomical location based on whether they involve the gastro-oesophageal junction. In 2014, Bass et al. proposed a classification system based on molecular characterisation with the aim of better stratifying patients for management and clinical trial design [3]. 

Adequate surgical resection is the only curative treatment for GC, however 50–70% of patients relapse after surgery [4]. Further, early-stage gastric cancer is often asymptomatic, and many patients present with advanced disease. First line chemotherapy for patients with metastatic disease consists most commonly of a combination of platinum compounds and fluoropyrimidine, which has only limited efficacy and the majority of patients experience disease progression. Recent advances are now permitting molecular characterisation of these tumours (reviewed in [5]), and clinical trials in appropriately selected patients investigating the use of combined chemotherapy with immunotherapy using inhibitors of HER2 [6] and PDL-1, for example have shown significant improvements in disease free survival. As a result, these are now recommended as standard of care [7]. A variety of inhibitors are also being assessed for third line chemotherapy and, in some cases significant benefits have been reported; however, the heterogeneity of results (reviewed in [8]) support the idea that in future better molecular classification of patients will permit better selection for targeted therapy. The identification of new targets will contribute to molecular characterisation, and bring us closer to effective personalized therapy for GC and improve on the current situation where three-year survival rates are as low as 20–30% even in high income countries [9].

An understanding of the mechanisms of cancer initiation would facilitate the development of new therapies and prevention strategies for GC. The main driving force for intestinal-type GC is chronic inflammation—triggered and maintained by one or more of the environmental factors mentioned above. In 1988, Correa [10] proposed the model where *H. pylori*-induced inflammation led to gastric atrophy and intestinal metaplasia, but the mechanisms were not readily understood. *H. pylori* infection is the highest single risk factor for developing gastric inflammation, while infection with a Cag positive strain is a strong risk factor for developing GC [11]. The capacity of *H. pylori* to establish a persistent infection establishes a scenario that can, over decades, overwhelm homeostatic processes, triggering metaplasia and DNA damage. 

Stem cell transformation is seen as a key step in cancer initiation—the mechanism may be due to direct changes, or inflammation-driven. After transformation has initiated development of a tumour, there is evidence that bone marrow-derived mesenchymal stem cells contribute to the maintenance of the tumour [12,13]. Our understanding of cell fate and stem cells in the stomach and intestine has gone hand-in-hand with the development of organoid culture systems. Organoid cultures—where primary cells derived from patients or experimental animals can be maintained in various formats—are emerging as excellent model systems to facilitate the study of cancer initiation. 

This review will focus on what we have learned using organoid cultures about the role of epithelial stem cells in the initiation of GC, the role of *H. pylori* virulence factors in triggering epithelial transformation, DNA damage and the links between chronic inflammation and transformation, with the stem cell as target. Some questions arising from recent studies will be discussed, and an outlook will be given on how organoids can now be used to address more complex questions; such as the role of the microbiota, the chronic inflammatory milieu, and how this affects the fibroblast-like cells of the mucosal stroma.

## 2. Gastric Homeostasis: The Key Role of Adult Stem Cells in the Gastric Gland

The gastric and intestinal epithelium are organised into glands with a crypt-like structure. These glands are one of a number of compartments that are constantly renewed throughout life, as part of the program of normal homeostasis. This process is now known to be directed by the activity of adult stem cells that are resident near the base of the gland [14,15]. The central control of the proliferation of these stem cells is managed by a system of signalling that orchestrates decisions about cell fate and migration. The wingless and -int proteins (Wnt) initially identified in Drosophila, have been shown to be pivotal to intestinal homeostasis (reviewed in [15]). Wnt3A mediates proliferation of intestinal stem cells to renew the crypt. Elegant fate-mapping studies have demonstrated that the intestinal stem cell doubles daily, and stochastically adopts either a stem cell, or transit amplifying (TA) fate [16]. TA cells are intermediate between stem and differentiated cells, and multiply several times before terminally differentiating. In the intestinal crypt, TA daughter cells differentiate into enterocytes, enteroendocrine and goblet cells, and migrate up the crypt where they are eventually shed into the lumen. In the intestine, some TA cells differentiate into Paneth cells and migrate to the base of the crypt [16]. 

**Figure 1:**
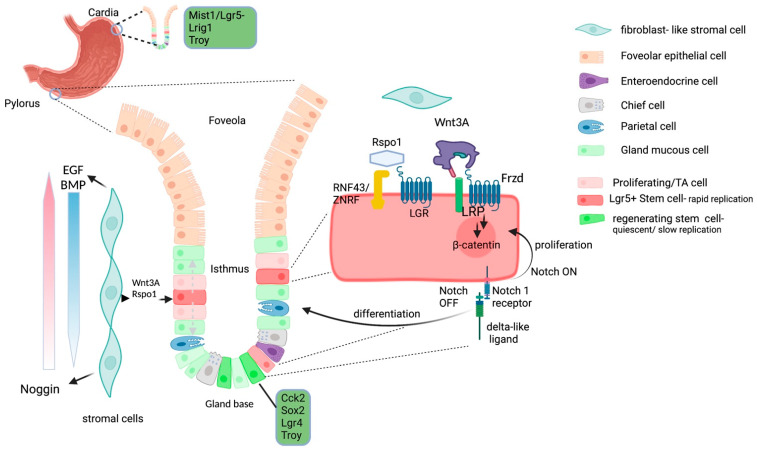
Overview of gastric homeostasis. A heterogenous population of adult stem cells maintain gastric homeostasis, resulting in a system of multiple back up-systems. Rapidly replicating Lgr5^+^ stem cells in the gland isthmus in the pylorus generate Transit Amplifying cells (TA) that differentiate in response to growth factors secreted by stromal cells, combined Notch and Wnt3A/B- Catenin and Rspo1 signalling control proliferation and differentiation decisions. Lgr5^+^ stem cells can replace the entire gland within 10–14 days. Wnt signalling in the absence of Notch leads to proliferation of the stem cell. Differentiation processes are controlled largely by EGF, and gradients of the growth factors Noggin and BMP. Mist1^+^ cells in the glands of the corpus respond to Wnt5 (and Rspo) signals and can replenish glands after injury. Lrig1^+^ and Sox 2^+^ cells can similarly respond. The Troy expressing population of quiescent cells are located at the base of the gland and are thought to act as a further reserve population. The combination of rapid and slower regenerative stem cells in the cardia and pylorus provides a system that is able to respond to damage, and acute and many chronic inflammatory insults. (Created with BioRender.com, 28 February 2022).

In the glands of the stomach, a number of different stem cells are present. In the mouse, stem cells have been identified both in the isthmus and the gland base [17] (Figure 1). Gastric pyloric stem cells divide to generate further stem cells, and TA cells that differentiate to replenish all cell types in the gland [18,19].

### Characteristics of Gastric Stem Cells

To date, a number of different cell populations have been identified as *bona fide* stem cells in the stomach. Originally identified in electron microscopy and pulse-chase experiments as granule free cells in the isthmus [20], cell-fate tracking studies carried out in transgenic mouse models and organoids have now identified stem cell markers including Lgr5^+^, Sox2^+^ cells in the pylorus, Cck2r^+^ in the antrum, and Mist 1^+^ [21,22], Sox2^+^ [23], and Lrig1 [24] in the corpus. Sox2, Lrig1 and RunX [25] define progenitors in both antrum and corpus. In addition, a “reserve” type stem cell expressing Troy has been identified in the corpus [26]. There is some evidence that these stem cells may express more than one of these markers. Investigations into the biological role of these stem cells in cancer initiation are shedding light on the complex environment that might lead to intestinal and diffuse type GC.

The most intensively studied population with regard to gastric homeostasis are Lgr5^+^ stem cells. The stem cells of the intestine, stomach, colon and hair follicle have been shown to express high levels of the leucine-rich repeat-containing receptor 5 (Lgr5^hi^) [14,18]. Lgr5 is expressed on the cell surface in a complex with the Wnt agonists Frizzled and low-density lipoprotein receptor-related proteins (LRP) [27]. Wnt signalling is in turn controlled by the transmembrane E3 ubiquitin ligases RNF43/ZNRF3 which are also part of this complex (Figure 1). Under homeostatic conditions, Frizzled is selectively ubiquitinylated by RNF43/ ZNRF3, resulting in degradation which effectively controls Wnt signalling [28]. The central role of Wnt is augmented by Roof plate specific spondin (R-spondin, Rspo) proteins which are produced by stromal cells and bind to Lgr5/LRP—binding of RSpo-1 to Lgr5 blocks the ubiquitination activity and permits Wnt signalling (largely Wnt 3a).

The cell fate decisions for differentiation or proliferation for Lgr5^+^ stem cells have been shown to be regulated by Notch protein signalling [29,30,31]. More recently, Wölffling et al. used human adult organoids to perform a detailed analysis of the growth factors required to differentiate different cell types in the gastric gland [32]. Bone marrow morphogenic protein (BMP) is known to suppress Wnt/β-catenin signalling [33], and BMP4 was shown to be expressed in the isthmus region in the human gastric corpus. Noggin is a BMP inhibitor and was expressed in the muscularis mucosae below the gland. Epidermal growth factor (EGF) is required for epithelial cell growth and is a component of standard organoid growth media. Wölffling et al. exposed mucosoid organoid cultures to different concentrations of EGF, BMP and Noggin to investigate differentiation. EGF was found to be a strict requirement for development of MUC5AC^+^ foveolar cells—deprivation of EGF and BMP permitted differentiation into chief cells, whilst deprivation of EGF and the presence of BMP promoted development of parietal cells. In the tissue, the spatial arrangement of cells expressing these factors establishes gradients that control differentiation (Figure 1). Changes in expression of these key factors are a mechanism for pathological alterations to the gland structure. 

The results of studies by Demitrack et al. reveal a more significant role for Notch signalling on Lgr5^+^ stem cell in the stomach than had been reported from the intestine [30]. This work led to the model where Notch signalling directly regulates proliferation of Lgr5 stem cells in antral glands [29,30,31]. Notch signalling occurs through close contact between Notch ligand (delta–like-DLL) on the surface of neighbouring cells. Active Notch signalling triggers proliferation of the stem cell, whereas inactive Notch favours differentiation [29,30,31]. In the intestine, this close contact is provided by Paneth cells [34]. In the stomach, mucous cells deep in the gland base express DLL-1 and were recently shown in a mouse organoid model to stimulate adjacent LGR5^+^ stem cells [35]. Thus, the Lgr5^+^ adult stem cell is a key target for inflammatory stimuli, increasing the risk of transformation. 

A recent study by Brischetto et al. [36] showed that strong NFκB activity was present in Paneth and Lgr5^+^ cells in the small intestinal crypt, and that NFκB activity was required for correct differentiation into Paneth and goblet cells, and thus plays a key role in small intestinal homeostasis. Interestingly, NFκB was essential for organoid growth. The ability of acute *H. pylori* infection to induce NFκB activation is well documented [37], if the observations discussed above are also true in the stomach, then the chronic NFκB activation seen in *H. pylori* infection, for example, provides a potential mechanism for dysplasia in the gastric crypt. 

Other studies on the control of epithelial proliferation revealed that Wnt signalling acting via Axin 2 could be detected in an additional population of Lgr5-cells [38]. This population was more proliferative than the Lgr5^+^ stem cells, and capable of repopulating the antral gland. Rspondin signals in Lgr5^−^/Axin 2^+^ stem cells are presumably mediated by Lgr4 which is expressed on epithelial cells, and have been shown to also respond to Wnt signalling [27]. These two stem cells provide a system of backups to facilitate repair, so the simple presence of *H. pylori* is not enough to initiate cancer.

The complex control of epithelial homeostasis is highlighted in a study by Hayakawa et al. [39], who identified a population of quiescent Mist 1^+^ stem cells located in the isthmus of the gastric corpus. These cells were able to give rise to cancer in both the corpus and the antrum. Later studies by the same group demonstrated that Mist 1^+^ cells respond largely to Wnt5a, and that the major source of Wnt5a is the innate lymphoid cell (ILC2) population [22]. The innate lymphoid population are the innate counterparts of T helper 2 responses that are typically associated with chronic *H. pylori* infection [40]. The effector and homeostatic function of ILC2 have been reported to be regulated by the gastric microbiota [41]. In a study of asymptomatic *H. pylori* infected patients, ILC2 were identified as the predominant ILC population in the stomach [41]. The predominance of this population in chronic infection provides a direct link between inflammation and cancer initiation through over-activation of Mist1^+^ stem cells. 

Similarly, Arnold et al. reported that Sox2^+^ adult stem cells were present in the gastric forestomach, corpus and pyloric crypts, and using cell tracing approaches, demonstrated that they are *bone fide* stem cells [42]. Later studies by the same group revealed that although this population are stem cells, Sox2^+^ cells were in fact dispensable for normal homeostasis. Sox2^+^ cells were susceptible to develop tumours in a mouse model with a mutation in Apc. Overall, it appears that while Sox2^+^ cells can act as slowly dividing stem cells, their primary action is as a tumour suppressor in the stomach by preventing Wnt/B-catenin signalling [43]. In an environment of chronic inflammation and/or in the presence of DNA damage or mutations which might enhance Wnt signalling, these cells can be seen as an important starting point for transformation. 

The role of gastric hormones in cancer initiation has been less well investigated, but cholecystokinin 2 receptor positive (CCK2R^+^) stem cells have been reported to be Lgr5 ^neg or low^ and located in the antrum. CCKR2 cells are located in the ^+^4 position, above Lgr5^high^ stem cells [21]. Interestingly, progastrin stimulation of CCK2R cells triggered their conversion into Lgr5^high^ cells, generating a larger pool of stem cells. Therefore, under conditions of chronic inflammation, or exposure to a carcinogen, overexpression of progastrin can therefore be a potential driver of carcinogenesis in the antrum. In 2018, lineage tracing studies revealed Lrig1^+^ cells as progenitor cells capable of repopulating glands in the corpus and antrum [24]. Lrig1^+^ cells are located in the isthmus and were able to repopulate the gland after acute injury. Experiments with Lrig1 ^null^ mice revealed that they are not however required for differentiation. This stem cell population may therefore act as a type of reserve to permit repopulation of the gland after injury.

Following the identification of Lgr5 as an adult stem cell marker, expression of Troy (coded by *Tnfrs19*) was found in intestinal stem cells [44], and also located at the base of the gland in the gastric corpus [26]. Fate mapping studies revealed that Troy1^+^ cells were capable of generating all stomach epithelial types with slower kinetics (gland replenishment in 1–3 months) than Lgr5^+^ cells (gland replenishment within 7–14 days). Troy^+^ cells appeared to be dispensable for normal homeostasis, and may act as a reserve population that is similar to the Lgr1 population—they may help repopulate the gland if the isthmus stem become damaged. Of further interest, cell sorting analysis of gastric glands revealed a population of Troy^+^ chief cells, which when cultured in vitro in organoid culture media were capable of generating organoids that could be differentiated into cells with transcriptional profiles that represent mucous neck or pit, but not enteroendocrine cells [26]. This result raises the concept of plasticity and challenges the long-established idea that cells can be terminally differentiated.

The heterogeneous nature of gastric stem cells as evidenced by the spatial distribution of, potential overlap and “reserve” role of some stem cells are now providing a foundation for us to understand the complexity of GC initiation. This goes some way towards providing a basis to explain why chronic immune simulation (e.g., as a result of chronic *H. pylori* infection, EBV infection, or auto immune gastritis) combined with specific virulence factors in the case of *H. pylori* infection, and host genetic and environmental (dietary) factors, act in combination to overwhelm the natural gastric homeostatic mechanisms.

## 3. Organoids as a Tool to Study the Role of Host Genetics in Gastric Cancer

Mechanistic connections between host genetics and gastric cancer phenotypes remain poorly understood, and cannot be addressed using animal models. To facilitate study of the role of genetic factors on growth control, Nanki et al. generated a bank of organoids from clinical samples taken from gastric cancer patients [45]. Expansion and maintenance of GC tissue in vitro required modifications of culture conditions to prevent overgrowth by normal gastric organoids. This was achieved by treating cultures with inhibitors to simulate signals that are dysregulated in human GC (i.e. TP53, RHO, TGF-β, RAS-PI3K). Using this approach, 37 patient-derived lines were generated. Analysis of responses of these organoids revealed that both genetic and epigenetic routes are relevant for the acquisition of independency to Wnt and Rspo [45]. Further, genetic manipulation of organoids using CRISPR approaches, confirmed the results of genetic analysis that compound mutations in CDH1/TP53 and RNF43/ZNRF3 were associated with a loss of dependence on Rspo, for example. A proportion of GC organoids grew without Wnt3A, and gene expression, genetic analysis and DNA hypermethylation studies showed that several mechanisms can lead to a lack of dependence on exogenous Wnt—self secretion, mutations in genes such as *APC*, and epigenetic regulation. Overall, this approach of generating organoids from human cancer tissues has provided links between genetic and epigenetic changes, and histological phenotypes observed in clinical practice, and a resource for further mechanistic studies. 

## 4. *H. pylori* Infection as a Direct Driver of Stem Cell Transformation

*Helicobacter pylori* infection is a potential initiator of cancer due to direct effects related to bacterial virulence factors, because it infects persistently and induces a chronic inflammatory state that is initially pro inflammatory, but over time develops a suppressive environment dominated by Treg in patients with subclinical infection [46,47,48].

To investigate direct effects of *H. pylori* on gastric stem cells, Boccellato et al. used mucosoids (2-dimensional air-liquid interface organoid cultures) from both mouse and human. Interestingly *H. pylori* were shown to form microcolonies deep in antral glands, and to interact directly with stem cells, triggering increased proliferation and activation of stem cells in the antral glands [49]. This is in keeping with the location of *H. pylori* in vivo in mice and based on analysis of biopsies. Thus, the organoid system reflects the in vivo scenario, and provides a direct link between bacteria and gland hyperplasia, a situation that increases the risk of transformation [49]. Of note is that this and two previous studies have shown that the bacterial effect was dependent on the presence of the CagA toxin, with isogenic ΔCagA mutants having reduced effect [50,51,52]. Additional studies investigating the role of CagA are discussed below. 

## 5. *H. pylori* Virulence Factors in Cancer Initiation

Investigations into the role of CagA in inducing signal transduction cascades in gastric epithelial cells were pivotal in our understanding of the early stages of *H. pylori* pathogenesis [53,54]. Translation of the observations made in AGS cells to the clinical picture, or to mouse models has been limited. Later studies in AGS, and MDCK cells revealed an intriguing role for the N-terminal region of CagA in driving structural changes to the apical complex [55]. The polarized nature and three-dimensional structure of gastric organoids has made it possible to study the mechanism by which the CagA virulence factor of *H. pylori* facilitates the disruption and polarization of the epithelial cells by mislocalizing and redistributing tight junction protein complexes on the apical side of the epithelium. Wroblewski et al. [51] reported that, compared to organoids infected with a CagA-negative mutant, murine gastric organoids infected with CagA-positive *H. pylori* 7.13 strain showed redistribution of tight junction proteins such as β-catenin and claudin 7—a tight junction protein that has been previously associated with other types of cancers [51]. Expression of claudin-7 was also reduced, and increased cellular proliferation was observed for the CagA positive strain, while organoids infected with the CagA negative mutant had similar levels to uninfected organoids. Inhibition of β-catenin in organoids prevented the cellular changes upon infection with the *H. pylori* strain, implying that this effect is β-catenin-dependent. Suppression of claudin-7 was also shown to be dependent on β-catenin and snail signalling in an MKN28 cell infection model, but a demonstration in organoids was not reported. 

In another study [56], binding of *H. pylori* CagA with apoptosis-stimulating protein of p53 2 (ASPP2) was reported to be responsible for the disruption of cell polarity via formation of a complex with the apical PAR complex-Par3 and PKC and the basolateral complex Par1. The colocalization of these protein complexes with CagA, and their redistribution from the apical tight junction to the apical side was observed in human gastric organoids infected with WT and a CagA mutant *H. pylori* for 24 hours. Furthermore, inhibition of the CagA-ASPP2 complex prevented the loss of cell polarity and reduced bacterial colonization. 

There have also been investigations into the role of virulence factor VacA. *H. pylori* VacA disrupts endolysosomal trafficking to cause gastric cancer and favours colonization. Capurro et al. [57] reported that this disruption by VacA is achieved via inhibition of transient receptor potential membrane channel mucolipin 1 (TRPML-1), an endolysosomal calcium channel. Initial experiments revealed that VacA creates intracellular space within the parietal cells of mouse *in vivo* which facilitates resistance to antibiotics and favours persistence in the host. VacA also inhibited TRPML-1 in AGS cells. Organoids generated from TRPML-1 deficient mice had vacuolations and accumulation of autophagosomes as well as intracellular compartments within the parietal cells containing *H. pylori* that persisted after eradication therapy. These results were reversed upon the activation of TRPML-1 by the administration of the synthetic agonist ML-SA1, resulting in functional lysosomes and reduced intracellular survival of VacA-positive *H. pylori*. In another study, Caston et al. [58] reported that, while both M1 and M2 variants of VacA have similar vacuolating activities in organoid cultures, they both increased cell vacuolation when interacting with the basolateral surface compared to interactions with the apical surface. Unlike the study by Capurro et al. [57] where all the experiments carried out in the organoids were replicated in mouse and gastric cancer cell lines, the differences in vacuolation between the apical and basolateral side in the latter study [58] would not have been possible without the use of organoids. 

### 5.1. H. pylori Induced DNA Damage

Gastric organoids have also aided the study of the mechanism by which *H. pylori* either induces DNA damage or favours the accumulation of DNA damage, resulting in gastric cancer. Sayed et al. [59] reported that, *H. pylori* infection suppresses the expression of Nei-like DNA glycosylase 2 (NEIL 2) in mouse organoids, independent of virulence factors such as *CagPAI* and VacA. NEIL2 is known to be involved in the repair of DNA damage by removing oxidised species [60]. The suppression of NEIL2 leads to the accumulation of DNA damage and can consequently result in gastric cancer. The anti-inflammatory effect of NEIL2 in *H. pylori* infection was also shown, as organoids generated from *H. pylori*-infected NEIL2 knockout mice had higher expression of proinflammatory cytokines compared to organoids generated from *H. pylori*-infected WT mice. This was replicated in mouse-derived gastric tissues and a further experiment revealed that DNA damage was higher in the NEIL2 knockout mouse. 

In a study in human-derived organoids, Bauer et al. [61] reported that DNA damage by *H. pylori* occurs in an ALPK1/TIFA/NF-KB-dependent manner in S-phase cells. The *H. pylori* LPS precursor (β-ADP-heptose) was sufficient to induce this damage. In this study, both human gastric organoids and AGS cells had a higher level of DNA damage and IL-8 production when infected with wildtype *H. pylori* compared to infection with *H. pylori* mutants lacking the rfaE enzyme which is responsible for the production of β-ADP-heptose, or CagPAI mutants, thus implying that this damage is rfaE and CagPAI dependent [61]. Similarly, Sierra et al. using a mouse model as well as organoids generated from both mouse and humans, reported that spermine oxidase (SMOX) enhances *H. pylori*-induced carcinogenesis by promoting inflammation, inducing DNA damage and activating the β-catenin signalling pathway [62]. 

### 5.2. Chromosomal Change

Organoids derived from dysplastic lesions from Lgr5-p53KO mice treated with *N*-methyl-*N*-nitrosourea MNU were generated in a recent study by Sethi et al. Dys-Lgr5-p53KO organoids had a modest increase in Lgr5 stem cells. The authors conclude that early p53 loss may confer renewal properties in gastric premalignancy [63]. When the chromosome count of dys-Lgr5-p53KO organoids was compared with lgr5-p53wt mice, the p53KO organoids had greater genome doubling. In addition, xenografts from the Lgr5-p53KO mice were capable of outgrowth. Somatic copy number (SCN) alterations were not observed in the organoids derived from dysplastic tissue; however the xenografts derived from them did. The results of this elegant study support the idea that organoids derived from premalignant tissues will facilitate the study of structural genomic changes. 

## 6. EBV as an Initiator of Gastric Cancer

A subset (9%) of gastric cancers are linked to Epstein Barr virus (EBV) infection—the cause of glandular fever/mononucleosis. Co-infection with *H. pylori* is an increased risk factor, but it is unclear which pathogen initiates the process. A recent study that compared EBV infectivity for matched normal and GC organoids generated from the same patient made an interesting observation—that EBV was only able to infect GC organoids, not normal organoids [64]. The EBV receptor ephyrin receptor A2 (EPHA2) is expressed on carcinoma cell lines, including the gastric carcinoma line AGS [65] but was not present on normal organoids [64]. This study raises questions about whether EBV can only infect epithelia after they have been transformed, or whether EPHA2 or another previously unidentified receptor may become expressed after chronic inflammatory activity. An alternative possibility is that EBV infected B cells which may infiltrate *H. pylori* infected cells (or otherwise inflamed gastric mucosa), permitting viral entry into epithelial cells [66]. Organoid cultures, and B cell co-culture systems provide a potential platform to address questions on the relationship between EBV and *H. pylori* in cancer initiation. Future studies in organoids generated from pre-cancerous tissues from patients with known genetic predisposition to GC could also be an interesting approach. 

## 7. Chronic Inflammation Creates a Milieu Balanced between Injury and Immune Suppression, Creating an Environment in the Stroma That Predisposes to Stem Cell Changes

**Figure 2:**
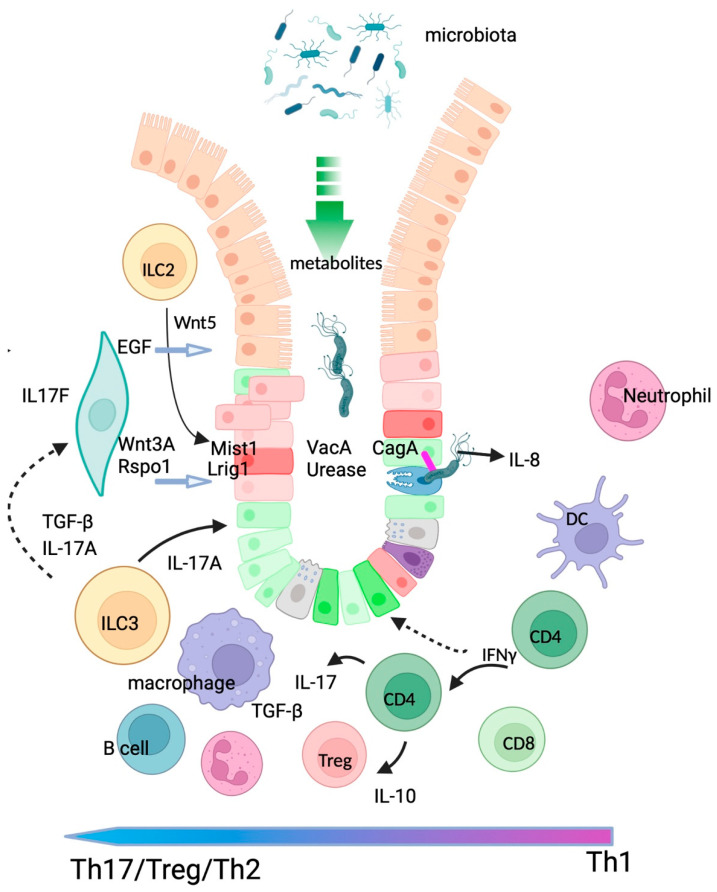
An environment of chronic inflammation provides an environment where immune and microbiota facts combine to potentially overwhelm gastric homeostasis and induce metaplasia. CagA^+^ strains of *H. pylori* trigger secretion of IL-8 by epithelial cells, attracting neutrophils and macrophages to the mucosa. Mucosal dendritic cells (DC) drive a CD4 T cell response which is not effective at clearing infection. Acute gastritis in characterised by Th1 cytokines including IFNγ which also promote atrophy. Over time, a strong Th2/Treg/Th17 response becomes established and controls damage. Predominant cytokines such as IL-17 are produced by both CD4+ T cells and ILC3 cells, IL17 acting in concert with TGF-β may stimulate stromal cells to overexpress growth factors such as Wnt3A, Rspo and EGF, leading to proliferation and differentiation of gland stem cells. Organoid and co-culture systems provide ideal experimental systems to investigate the role of inflammatory state on cancer initiation. (Created with BioRender.com, 28 February 22).

It is widely accepted that chronic inflammation provides predisposing conditions for transformation; however the events that link this to cancer initiation are only partly understood. The best characterised predisposition to GC is caused by infection with *H. pylori*. The triggering of innate inflammatory events after interaction of *H. pylori* with gastric epithelial cells has been well studied in vitro ([67] for review) and in organoid models as discussed above. Persistent infection, acute and long-term inflammation have also been studied extensively in animal models (reviewed in [68]), and in *H. pylori* positive patients [69]. Acute *H. pylori* gastritis is characterised by secretion of IL-8 and is followed by Th1 biased responses and infiltrations of neutrophils, macrophages and T-cells into the gastric mucosa [70]. Over time (i.e., years to decades) the combined effects of bacterial virulence factors, and immune evasion strategies results in establishment of a persistent infection which is biased toward Th2/Th17/ Treg (Figure 2) [reviewed in [71]]. The development of gastritis, ulcer, and metaplasia are thought to result from a failure to control chronic inflammation. For example, patients with *H. pylori* gastritis had high numbers of CD4^+^ and FoxP3^+^ T cells, and higher levels of expression of TGF-β, IL-10 than patients with peptic ulcer where Treg numbers were reduced [72] consistent with reports from earlier studies [73]. The question of why only a small proportion of *H. pylori* infected individuals develops symptoms remain largely unexplained but is linked to host genetics, and the virulence factors of the infecting strain.

An interesting recent survey of *H. pylori* infected patients who were asymptomatic used single cell RNA sequencing and revealed that infected patients had expanded CD4^+^ (including Treg and Tfh populations) and B cell populations, reduced CD8^+^ CDllc^+^ myeloid cells, and innate lymphoid cells biased toward the NKp44^+^ ILC3 type [69]. The study also addressed questions on the role of the microbiota as discussed below. The presence of a strong Treg population and high levels of IL-17 are consistent with the model of a tightly controlled environment of regulations and “damage control” that prevents most patients from developing severe outcomes. 

Interleukin 17 is emerging as a key cytokine of interest in chronic inflammation, including in *H. pylori* gastritis and cancer (Figure 2). Receptors for IL-17 are expressed on a range of cell types for example, IL17RA is chiefly expressed in immune cells, whereas IL17RC is expressed on non-lymphoid cells such as epithelial cells. While IL-17A clearly plays a key role in protecting mucosal surfaces from bacterial, helminth and fungal infection [74], it has been reported to have both pro- and anti-tumorigenic roles in cancer (reviewed in [75]). Interestingly, IL-17 has been shown to be a relatively weakly activating cytokine, and that it acts in concert with other cytokines such as IL-6 and TGF-β [76], which is also predominant in *H. pylori* gastritis (58), and EGFR [77]. Notably, in a recent study, IL-17A-mediated activation of EGFR was critical for activation and migration in Lrig1^+^ stem cells in a skin wound healing model [77]. If similar events occur in gastric tissue, this would provide direct links between a chronic inflammatory microenvironment and stem cell function.

It seems likely that under conditions of chronic inflammation that gastric stromal cells may become responsive to inflammatory cytokines, which in turn can drive changes in secretion of stromal growth factors such as Wnt and RSPO. Indeed Mucclio et al. [78] reported that fibroblasts from IL17A^−/−^ mice were able to prevent tumour invasion in a pancreatic ductal adenocarcinoma (PDA) model. The effects in this study were attributed to increased levels of expression of IL-17F in the affected mice [78]. Further, individuals with polymorphisms in IL17A (G197A) or IL17F (T7488C) have significantly increased risk of gastric cancer [79]. If gastric stromal cells respond in a similar manner, it could provide a mechanism for inflammation induced changes to homeostasis. Similarly, the effects of anti-inflammatory Treg cytokines such as IL-10 on the stroma should be investigated. 

Organoid models such as the mucosoid systems described by Boccellato et al. [49] would be ideal to investigate the effects of cytokine milieu directly on stem cell proliferation (*via* IL17RC expression on epithelia, for example). The effects of cytokines on growth factor secretion by gastric stromal fibroblasts-like cells could also be addressed in relatively simple 2-D in vitro models. The possibility for Wnt5a as an additional factor in stem cell proliferation was raised by Nienhuser et al. [22]. An additional source of growth factors may be the innate lymphoid population and the contribution for Wnt5. Co-culture of CD4^+^ T cell and ILC populations isolated from cancerous and pre-cancerous mice with mucosoid cultures could be used in combination with fate mapping approaches to address questions on how Wnt and Notch signalling in stem cells are affected.

## 8. Organoid Cultures as Tools to Study Progression from Metaplasia to Gastric Cancer

Organoids have also been useful in the study of the progression from gastritis to gastric cancer characterised by parietal cell loss and development of spasmolytic polypeptide/Trefoil Factor (TFF) 2-expressing metaplasia (SPEM) in its early stages [80,81]. A high proportion of gastric cancer cells express programmed death ligand 1 (PD-L1), an immune-suppressive ligand that is known to suppress the function of effector T-cells [82,83]. Holokai et al. investigated the role of the Sonic Hedgehog (Shh) signalling pathway in PD-L1 expression using gastric organoids and an organoid-cytotoxic T-lymphocytes (CTL) co-culture model [84]. When human gastric organoids generated from both the antrum and fundus were microinjected with the *H. pylori* G27 strain, they were found to develop dysplasia as determined by the over-expression of SPEM markers such as Trefoil factor 2 (TFF2) and *Griffonia simplicifolia* II GSII (lectin binding GlcNac mucin residues), as well as the gastric cancer stem cell marker CD44v9. In the study, human gastric organoids infected with CagA-positive and negative *H. pylori* G27 mutant had increased expression of PD-L1 in a CagA-dependent manner compared to uninfected controls. Pretreatment with Hedgehog/Gli inhibitor-GANT61, prevented PD-L1 expression, revealing that this response is Shh-mediated. 

Cytotoxic T-lymphocytes are the main inductors of apoptosis in gastric cancer cells [85]. To study the effect of the interaction between PD-L1 and PD-1 on CTL proliferation and cell death during *H. pylori* infection, organoids were co-cultured with autologous CTL and/or dendritic cells. *H. pylori* infection led to reduced CTL proliferation and increased PD-L1 expression on epithelial cells. Pretreatment with a PD-1 inhibitor decreased PD-L1-expressing epithelial cells and increased CTL proliferation. Overall, the expression of PD-L1 on SPEM cells was found to be dependent on Shh signalling, and the interaction of PD-L1 with PD-1 on CTLs enabled the survival of SPEM cells in the presence of *H. pylori* infection. This finding opens potential avenues to develop targeted therapies for patients with metaplasia where eradication of *H. pylori* infection is no longer beneficial.

In another study Osaki et al. [86] reported that IFN-γ mediates the progression from gastritis to atrophic gastritis and metaplasia—which are preliminary stages of gastric cancer. Corpus-derived organoids were used to show that the gastric epithelial cells express receptors for IFN-γ. Treatment with IFN-γ resulted in the death of the organoids in a receptor-dependent manner. Additionally, treatment of organoids with supernatant from the lymph nodes of a mouse model of autoimmune gastritis-TxA23 also resulted in cell death compared to organoids treated with supernatants from IFN-γ knockout mice. Further investigation in mouse tissues by assessing the early-stage features of gastric cancer such as parietal cell loss (atrophy), mucus neck cell hyperplasia and development of metaplasia (SPEM) that occurs during the progression to gastric cancer showed that, compared to WT, TxA23 mice deficient in IFN-γ lacked hyperplasia, had more healthy parietal cells, and minimal mucus neck cell hyperplasia [86]. 

Similarly, in studies that combined the use of organoids with animal models, Bertaux-Skeirik et al. reported that cluster of differentiation gene (CD44) while acting as a coreceptor for c-Met, influences the proliferation of epithelial cells [87]. Compared to CagA mutants, fundic gastric organoids generated from human or mouse and infected with the *H. pylori* G27 strain had increased proliferation of epithelial cells with a corresponding increase in the expression of phosphorylated c-Met which coprecipitates with CagA [87]. They also showed an epithelial-to-mesenchymal phenotype. Pretreatment of human fundic gastric organoids with either neutralising antibody to CD44v6 or c-Met inhibitor reduced the proliferation. In vivo inhibition of CD44 in Mongolian gerbils prevented the development of atrophic gastritis. Thus, organoids are not only useful for the study of early stages of gastric cancer, but can also be used in identifying potential therapeutic targets.

### Organoids as Tools for Loss of Function Studies

Organoids have also helped in the discovery of antitumorigenic proteins where loss of function increases the rate of gastric cancer development. Intracellular Nod1 recognises *H. pylori* peptidoglycan delivered by the CagT4SS and can increase the inflammatory response in *H. pylori* infection [88]. In a study to investigate the role of Nod1, organoids generated from Nod1 knockout mice had increased expression of NF-KB target genes compared to WT C57BL/6 mice after infection with *H. pylori* strains PMSS1 and 7.13 for 6 and 24 hours [89]. To further assess the effect of *H. pylori* infection on cytokine production by innate immune cells, epithelial gastric organoids from both infected WT and Nod1 KO C57BL/6 mice were cocultured with macrophages from the same mice. Increased cytokine production was observed in Nod1 deficient samples, especially when both the macrophages and gastroids lacked Nod1. In vivo investigation in both C57BL/6 and INS-GAS mice (known to be more prone to gastric cancer development) further confirmed that loss of Nod-1 accelerates the development of cancer and cytokine production. Thus, the authors concluded that functional Nod-1 is necessary for tumour suppression. 

## 9. The Gastric Microbiota Intersects with the Inflammatory Response and Gastric Homeostasis

The link between diet and gastritis has long been accepted. While diets high in salt and other preservatives have been linked to carcinogenic effects, diet also influences the diversity of the microbiota at all levels of the gut [90,91]. *Helicobacter* infection has been reported to result in reduced diversity in the gastric microbiota in mouse models [92], gerbils [93], and in asymptomatic infected individuals [69]. While in GC patients, differences were noted between early and advanced gastric cancer in terms of microbiome [94]. A recent meta-analysis of 14 clinical studies concluded that gastric dysbiosis occurs in gastric cancer, with reduced diversity and a decline in the microbiota population [95]. 

To investigate the links between microbiota and immune response Satoh-Takayama et al. [41] observed that germ-free (GF) mice had significantly reduced proportions of ILC2 cells, suggesting that this population are induced by the intestinal microbiota. When GF mice were reconstituted with microbiota, ILC2 numbers recovered. Levels of ILC2 correlated to levels of IL-7, and further analysis revealed that mice treated with vancomycin, but not other antibiotics, had reduced levels of ILC2, and metagenomics analysis linked the S24-7 (*Muribaculaceae*) group of the *Bacteriodetes* with development of ILC2 [41]. The S24-7 group have not been extensively studied, but were correlated with propionate production in a recent study [96].

The critical role of metabolites, particularly short-chain fatty acids (SCFA) produced by the microbiota have been extensively investigated in mice and in humans [97]. Using mouse models and in vitro assays, Yang et al. have found that SCFA-induce cytokine production by both ILC and T helper cells in the lamina propria via GPR41, triggering signalling events that lead to expansion of the ILC2 and ILC3 type which promote homeostasis and repair [98] (Figure 2). Extension of these studies by utilizing organoid cultures would permit investigations on any effects of bacterial metabolites on gastric stem cells. 

## 10. Conclusions and Future Directions

Recent years have seen great advances in our understanding of gastric homeostasis and the control of the adult stem cells. A great deal of this work was made possible only with the use of organoid cultures of various types. This approach has also permitted systematic comparisons to be made of mouse and human tissues, and has provided insights into the roles of growth factors, and different stem cell types in the corpus and pyloric regions, which goes some way towards explaining the heterogeneity of GC.

Generating organoid cultures from normal, infected or cancerous human gastric tissue has permitted insights into the role of growth factors, and made possible detailed analyses of genetic changes. It has also provided cell lines that can be cultured long-term. It is of note that organoid cultures established from different regions of the stomach (corpus or antrum) retained gene expression patterns typical of the source; this further supports the idea that the stem cells in each region are different [32], or perhaps that the assemblage of stem cells expressing different markers (Mist1, Lrig1, Lgr5, Sox, for example) are characteristic of the region. 

Mechanistic questions about how an inflammatory milieu impacts gastric stem cells can now be addressed. It is possible or even likely, that the different stem cell types respond differently to local inflammatory stimuli, although future studies will need to address these more complex relationships. 

Armed with an in-depth knowledge of gastric homeostasis, and of the mechanisms by which *H. pylori* virulence factors can impact epithelial and stem cell function [97], we are now in a position to investigate further gaps in our understanding, e.g.,: of how the chronic inflammatory state can lead to GC induction; the interplay between the microbiota, gastric homeostasis, and inflammation; and to investigate how chronic inflammation impacts the stroma—a population that has been largely ignored to date. Similarly, there are knowledge gaps regarding the role of gastric hormones in an environment of disrupted gastric homeostasis.

To tackle these questions, we propose that the complexity of organoid model systems will need to increase to include co-cultures with immune cell populations, and/or cytokines. Similarly, the inclusion of myofibroblasts in more complex models will permit detailed analyses of their response to inflammatory stimuli. Recent studies have underlined the importance of growth factor gradients for gastric homeostasis [32], and we predict that cytokine gradients will also be key to understanding the effects of cytokines on gastric stem cells. To this end, controlled-release delivery using nanoparticles or other engineered systems to generate gradients in vitro will be advantageous (see for example [99]).

Such models will provide a platform to systematically investigate the effects and interplay of inflammatory and regulatory cytokines, and cell populations on homeostasis and cancer initiation in the gastric gland. The genetic manipulation of organoids also promises the opportunity to study the role of specific factors in cancer initiation under controlled conditions without the possible compensatory effects in gene-deleted mice. 

A caveat that must be considered for studies using in vitro organoid models to date is that the role of microbiota has not been addressed. There is great scope to use these systems to address questions on the specific effects of bacterial metabolites, particularly SCFA on different cell types in the gastric gland, directly, and also in more complex configurations where immune cell populations could be introduced. The evidence linking microbiota, innate lymphoid cells and secretion of growth factors is of particular interest. Analyses of the microbiome in patients with established GC provides us with evidence of the impact on the microbiota after the fact. Organoid culture systems based on normal stomach tissues now provide an opportunity to dissect the roles of bacterial metabolites on the early stages of cancer initiation. 

As discussed above, stromal cells are the source of growth factors that regulate homeostasis, and therefore stem cell proliferation. Dissection of the response of stromal cells such as fibroblasts to the inflammatory milieu established by chronic *H. pylori* infection will not only close the circle of our understanding of gastric inflammation, but will also provide potential targets for therapeutics and vaccines.

## Data Availability

Not applicable.
